# Effect of C-2 substitution on the stability of non-traditional cephalosporins in mouse plasma

**DOI:** 10.1038/s41429-019-0167-y

**Published:** 2019-03-22

**Authors:** Matthew Zimmerman, Stacey L. McDonald, Hsin-Pin Ho-Liang, Patrick Porubsky, Quyen Nguyen, Cameron W. Pharr, Andrew J. Perkowski, Robert Smith, Frank J. Schoenen, Ben S. Gold, David Zhang, Carl F. Nathan, Véronique Dartois, Jeffrey Aubé

**Affiliations:** 1 Public Health Research Institute, New Jersey Medical School, Rutgers, the State University of New Jersey, Newark, NJ 07013 USA; 2 Division of Chemical Biology and Medicinal Chemistry, UNC Eshelman School of Pharmacy, University of North Carolina at Chapel Hill, Chapel Hill, NC 27599 USA; 3 Specialized Chemistry Center, University of Kansas, Lawrence, KS 66045 USA; 4 Department of Microbiology and Immunology, Weill Cornell Medicine, New York, NY 10065 USA

**Keywords:** Pharmaceutics, Medicinal chemistry

## Abstract

A systematic study of the stability of a set of cephalosporins in mouse plasma reveals that cephalosporins lacking an acidic moiety at C-2 may be vulnerable to β-lactam cleavage in mouse plasma.

## Introduction

**Fig. 1:**
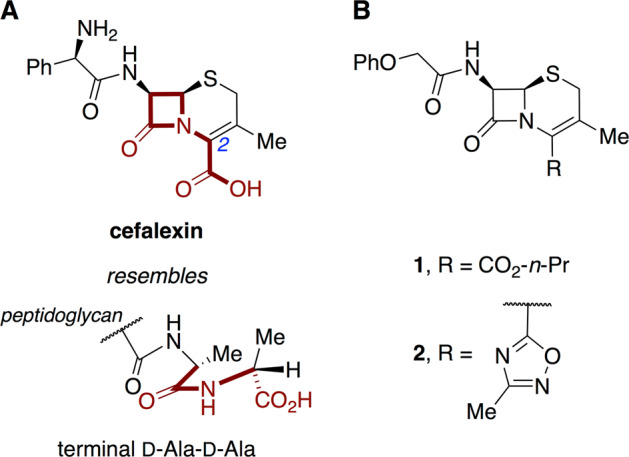
Structures of **a** cefalexin, a clinically used cephalosporin, and the generally accepted pharmacophore for cephalosporin antibiotics, and **b** two cephalosporins shown to be active in culture against *Mtb* [4]

The β-lactam antibiotics, such as the penicillins and cephalosporins, are among the most storied therapeutic agents in medicine. The most common mechanistic targets for these agents are the penicillin-binding proteins (PBPs), which are D,D-transpeptidases critical for cell-wall peptidoglycan synthesis in bacteria [1–3]. A typical cephalosporin, such as cefalexin, binds to and inhibits the action of PBPs because it resembles the structure of the terminal end of a crosslinking peptide chain, D-Ala-D-Ala (Fig. 1a). Accordingly, the pharmacophore of β-lactam antibiotics is considered to comprise both the β-lactam ring (which is strained and therefore contains an electrophilic carbonyl for attack by the PBP) and the C-2 carboxylic acid, which mimics the C-terminal acid of the native peptide chain.

We recently reported two sets of cephalosporins that lack the C-2 carboxylic acid of the class but instead bear a neutral ester (**1**) or oxadiazole (**2**) group at this position (Fig. 1b) [4]. These compounds are active against *Mycobacterium tuberculosis* (*Mtb*) under non-replicating conditions. This is significant because there are only a few reported cases of β-lactams being active against tuberculosis [5–8]. In seeking to pursue advanced studies of compounds **1** and **2**, we determined that the compounds were unstable in mouse plasma, thus prohibiting initial murine experimentation. In this paper, we report evidence that the locus of this instability is β-lactam hydrolysis correlated with the presence or absence of an acidic substituent at C-2 of compounds examined.

## Materials and methods

### Chemistry

Most new compounds in this study were prepared as previously reported for compound **1** [4]. Thus, amide bond formation and esterification of the widely utilized starting cephalosporin core 7-aminodesacetoxycephalosporanic acid (7-ADCA) provided *N*-acylated cephalosporins whereas C-2 esters were prepared through subsequent esterification. The tetrazole-substituted cephalosporin **7** was prepared as described in the following section. See Supplementary Information for experimental details and characterization data.

### Chemical stability

Compounds (5 μg/mL) were suspended in media (1:1 PBS/acetonitrile) at room temperature and pH = 5 and examined by HPLC/MS every 12 h for 6 days. Data are reported as percentage remaining in the supernatant as compared to time t_0_. Experiments were carried out in duplicate.

### Plasma stability

The stability assays were carried out using plasma from female CD-1 mice (Bioreclamation). Stability samples consisted of 5 μL of stock compound solution in 100% DMSO and 95 μL of plasma to a final 10 μg/mL concentration. The samples were incubated at 37 °C with shaking; 10 μL were removed at each time point and combined with 100 μL of 1:1 acetonitrile/methanol, 10 ng/mL of verapamil, and 10 μL of acetonitrile/water. Samples were analyzed by LC-MS/MS using the Q-Exactive high-resolution mass spectrometer (Thermo Fisher Scientific). Stability was calculated as the percent remaining of the parent compound compared to the initial concentration otherwise. Stability in marmoset plasma was similarly carried out.

### **Antimicrobial assays**

Antimicrobial assays were carried out as reported [4].

## Results

**Fig. 2:**
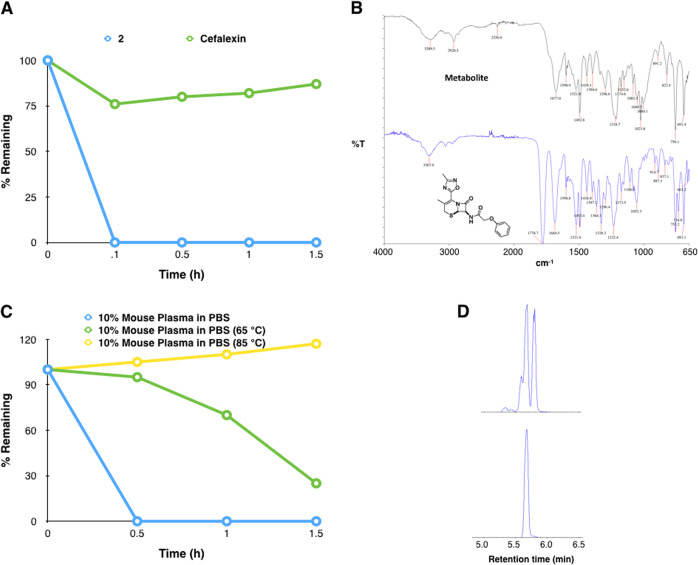
Results obtained by treating cephalosporin **2** with mouse plasma. **a** Comparison of overall stability of cefalexin and **2** in mouse plasma. **b** Evidence that the β-lactam ring is cleaved by incubating a sample of **2** in mouse plasma. Top: IR spectrum obtained by incubating a small amount of **2** with mouse plasma and isolating a small amount of product. Bottom: IR spectrum of **2**. **c** Effect of pre-heating plasma prior to incubation of compound **2**. **d** Portion of HPLC traces obtained by chemically hydrolyzing compound **2** via heating at pH = 14 (top) or by treatment in mouse plasma as in panel A. (Note: this figure has been edited for clarity and space; see Figure S1B in the Supplementary Information for the full HPLC traces)

As reported [4], compound **2** had good chemical stability in buffer pH of 7.4 but was rapidly degraded in mouse plasma, with <5% of the initial concentration of compound found remaining after 5 min incubation (Fig. 2). For comparison, cefalexin is relatively stable in mouse plasma over 1.5 h. MS analysis of the analyte showed that the metabolite added 18 atomic mass units (amu) to the parent mass, suggesting β-lactam hydrolysis. This was confirmed by carrying out the incubation on “preparative” scale (ca. 2 mg) and obtaining an IR spectrum of the product, which clearly showed the disappearance of the distinctive β-lactam ν_C=O_ stretch at 1779 cm^–1^ (Fig. 2b; this product was not obtained in sufficient quantities for full characterization, although the NMR was consistent with that of the ring-opened analog of compound **2** (Supplementary Information, Figure S1A)).

Two lines of experimentation were pursued to test whether the observed β-lactam cleavage arose from simple chemical hydrolysis or might be attributed to an enzymatic factor present in mouse plasma (Fig. 2c). In the first, the stability of compound **2** was compared across three plasma samples. One such incubation was carried out at room temperature as previously described. In the other two, the plasma was pre-heated at 65 and 85 °C, respectively, to determine the effect of possible heat-caused denaturation on the hydrolytic capability of the plasma. The plasma was cooled to rt prior to incubation with the cephalosporin. Comparison of the three experiments clearly indicated that the compound was more stable when treated with heated plasma, to the degree that no decomposition at all was observed for the sample pre-heated at 85 °C. In addition, although these cephalosporins are chemically stable in solutions of moderate pH, they do decompose when heated under strongly acidic or basic conditions (pH 1 or 14, respectively). When so challenged, multiple products were obtained, presumably resulting from epimerization, double bond migration (known to occur in cephalosporins [9]), or other transformations. Comparison of the HPLC traces from a sample forcibly destroyed at high pH with those subjected to simple treatment with PBS/mouse plasma clearly indicates that the latter is a significantly cleaner reaction, also consistent with relatively mild conditions accompanying a possibly enzymatic β-lactam cleavage (Fig. 2d). We note that carrying out the incubations in the presence of the esterase inhibitor NaF did not affect the rapid degradation of these compounds.

**Fig. 3:**
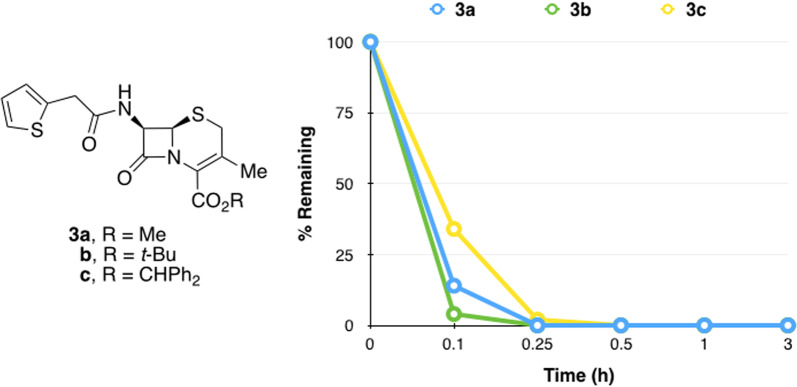
Effect of ester structure on stability of C-2 cephalosporin esters **3a**–**c**

The MS data from the incubation of C-2 *n*-propyl ester-containing **1** showed that the primary metabolite also had a mass of 18 amu higher than that of the starting cephalosporin, suggesting that β-lactam cleavage occurred in this case as well, rather than ester hydrolysis (which would result in a change of –60 + 18 = –42 amu). In fact, the 1,2,4-oxadiazole—an established ester bioisostere [10, 11]—has been initially prepared on the assumption that ester hydrolysis would occur in vivo for compound **1**, so the apparently preferential reaction of the lactam was surprising. To determine the effect of the nature of the ester on this feature, the stability in mouse plasma of a series of cephalosporins containing increasingly bulky esters (**3a**–**c**) was compared (Fig. 3). In each case, quick decomposition to afford the ring-opened amino acid was observed, with only modestly longer lifetimes noted for the diphenylmethyl ester.

**Fig. 4:**
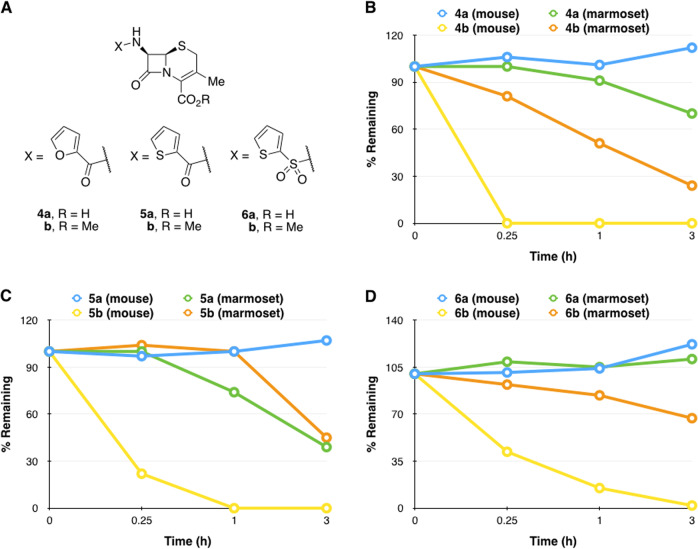
Stability comparison of acid/ester pairs in mouse and marmoset plasma. **a** Chemical structures and stability of (**b**) **4a**, **b**, (**c**) **5a**, **b**, and (**d**) **6a**, **b**

In our previous work, compound **2** was found to be considerably more stable in human than mouse plasma [4]. Thus, to determine the generality of the above observations and specifically interrogate the species dependence of the effect, three additional pairs of acid- and ester-containing cephalosporins **4**–**6** were prepared and examined for stability in both mouse and marmoset plasma, chosen because antitubercular drugs are commonly studied in marmosets [12, 13] (Fig. 4). As before, most of the compounds bearing a carboxylic acid at C-2 (series **a**) were stable during the entire time course of the experiments, regardless of whether they were performed in mouse or marmoset plasma (although **5b** seems to be degraded to a larger extent under these conditions). In contrast, the corresponding esters (series **b**) were rapidly converted to the amino acids resulting from β-lactam cleavage. Most interestingly, all three esters were substantially more stable in marmoset plasma when compared to mouse plasma, with as much of 70% of the parent compound still being detectable for **6b** after 3 h.

**Fig. 5:**
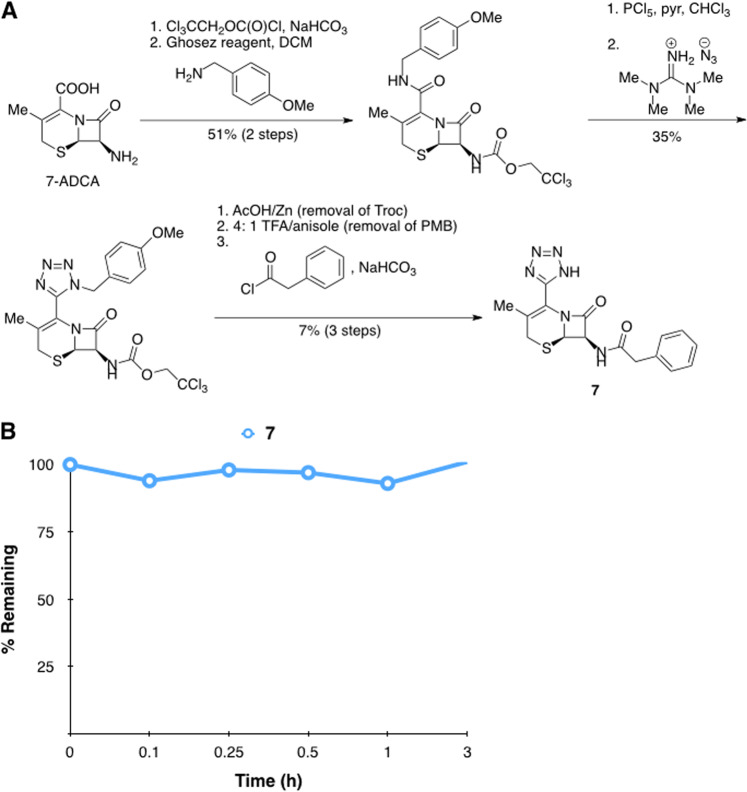
**a** Synthesis and **b** stability of tetrazole **7**

Finally, it was of interest to determine whether the apparent stabilizing power of the carboxylic acid at C-2 was specific to carboxylic acids per se or whether other acidic groups might behave similarly. Accordingly, we prepared the C-2 tetrazole-containing cephalosporin **7** as shown in Fig. 5a, driven in part by the hope that the tetrazole, beyond acting as a carboxylic acid isostere, would resemble the 1,2,4-oxadiazole moiety enough to confer anti-TB activity onto the compound. Compound **7** proved somewhat challenging to prepare, but was made following a procedure reported by Barth [14], first protecting the primary amine of 7-ADCA with a trichloroethoxycarbonyl (Troc). Using Ghosez’s reagent [15], we converted the C-2 carboxylic acid to the corresponding acid chloride, which was then converted to the *p*-methoxybenzyl (PMB) amide. In the key step, this amide was transformed to the PMB-protected tetrazole by converting the C-2 amido group to the corresponding iminyl chloride and subsequent cyclization using tetramethylguanidinyl azide [14]. Successive removal of the two protecting groups and amidation afforded **7** in low yield but in sufficient quantities for stability measurements and examination of its activity against *Mtb*. As is often the case, there was good news and bad news. The good news was that the tetrazole conferred consummate stability onto the cephalosporin; no degradation was observed during the entire time course of the experiment (Fig. 5b). Unfortunately, tetrazole **7** was completely bereft of activity against *Mtb* under either replicating or non-replicating conditions (data not shown).

## Discussion

The stability of cephalosporins and other classes of β-lactam antibiotics in biological media is necessary for the preclinical testing in relevant animal models. In particular, determination of protection against infection in mice is a very common early step in antibiotic development, including that of anti-TB agents [16]. Accordingly, the lack of stability of some cephalosporins lacking the usual C-2 carboxylic acid in mouse plasma presented us with an unexpected barrier to the development of compounds found active in cellular assays. Minimally, the results obtained in this study represent a caveat to those seeking to study non-traditional cephalosporins as potential therapeutic agents.

**Fig. 6:**
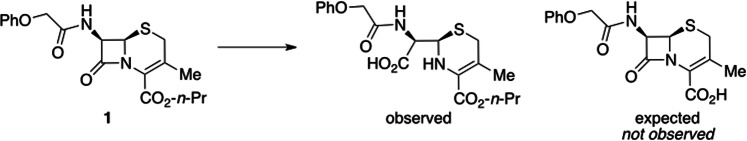
Observed and expected products of decomposition of cephalosporin **1** in mouse plasma. All compounds drawn in neutral form

Most surprising was the specific nature of this instability. Early in our studies, we uncovered the *n*-propyl ester **1** as active against *Mtb* under nonreplicating conditions [4]. In contemplating further studies in this compound, we immediately focused on the possibility that the ester group would undergo hydrolysis in vivo, thus generating the parent acids known to be inactive against our target. This led us to undertake to the design and synthesis of the 1,2,4-oxadiazole **2** as a bioisosteric replacement specifically to avoid this problem. We were therefore surprised to see that both the oxadiazole and ester were unstable in mouse plasma, especially when the locus of instability in each set of compounds was determined to be the β-lactam and not the ester (Fig. 6).

Cephalosporin C-2 esters have regularly been reported in the literature as prodrugs, commonly as germinal carboxylates [11, 17–21]. For at least one C-2 methyl ester, β-lactam hydrolysis was reported under some conditions [22]. Clearly, the observation of biological activity in many of these cases shows that ester instability does not always precede β-lactam opening and drug inactivation. However, our results do suggest that care should be taken to test for β-lactam inactivation under some circumstances. Along these lines, we note that we have observed similar instability in many cephalosporins containing a neutral group at C-2 beyond those reported here.

We do not currently know the causative agent for this β-lactam ring opening. However, several lines of circumstantial evidence lead us to speculate that some enzymatic factor in mouse plasma is responsible. These include the chemical stability of the cephalosporins under the conditions noted, different product profiles obtained from chemical vs. plasma-mediated opening, increased stability when plasma is pre-heated, and differences obtained when incubating compounds in plasma derived from different species. The latter consideration is especially noteworthy, since it suggests that stability in mice, a very common model of antibiotic function, may not fully predict a compound’s behavior in the ultimately desired therapeutic setting.

The chemical cause of this instability is likewise unknown, but all of our evidence to date suggests that the presence of a charged moiety at C-2 serves a protective function against this observed instability. The most compelling example of this is the observation that the C-2 tetrazole-containing **7** is stable under these circumstances. At this point, we hypothesize that whatever factor is responsible for the observed instability does not accommodate a charged group in its active site or that the charge per se stabilizes the β-lactam linkage by electrostatically disfavoring nucleophilic attack onto the lactam carbonyl group.

In summary, a systematic study of the stability of a set of cephalosporins in mouse plasma reveals that cephalosporins lacking an acidic moiety at C-2 may be vulnerable to β-lactam cleavage in mouse plasma.

## Supplementary information

Supplemental Information
