# Supplementary material for: Effect of C-2 substitution on the stability of non-traditional cephalosporins in mouse plasma
Source: J Antibiot (Tokyo). 2019 Mar 22;72(6):469–75. doi: 10.1038/s41429-019-0167-y (PMC7255492; doi:10.1038/s41429-019-0167-y)
Supplement: Supplementary file 1 — Supplemental Information [file 41429_2019_167_MOESM1_ESM.pdf]

Effect of substitution on the stability of non-traditional cephalosporins in mouse plasma

Matthew Zimmerman, Stacey L. McDonald, Hsin-Pin Ho-Liang, Patrick Porubsky, Quyen Nguyen, Cameron W. Pharr, Andrew J. Perkowski, Robert Smith, Frank J. Schoenen, Ben Gold, David Zhang, Carl Nathan, Véronique Dartois, and Jeffrey Aubé

Supplementary Information

Figure S1 .... S-1

Chemistry S-2

A

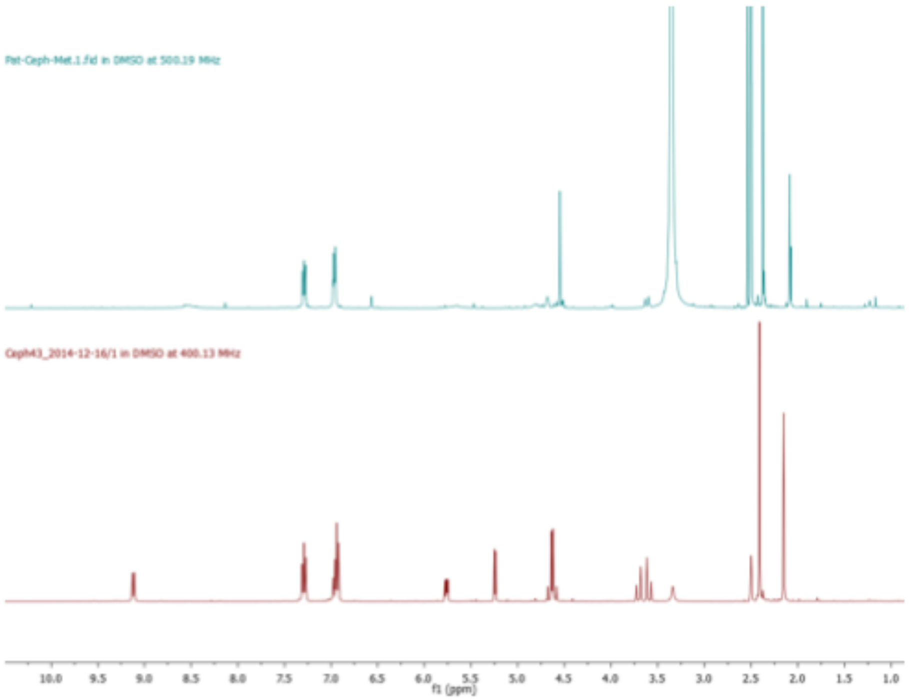

B

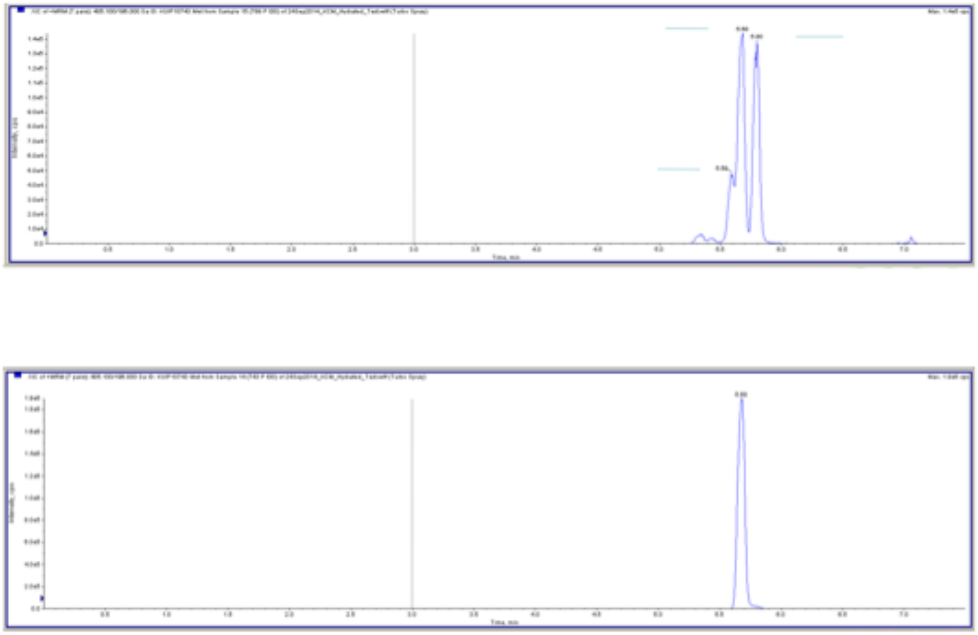

FIgure S1. Data from “preparative” incubation of cephalosporin **2** in PBS/mouse plasma. (A) A sample of the compound was treated with 50% PBS/mouse plasma at neutral pH for 1 h, partitioned between DCM and water, and the organic layer dried and analyzed by NMR. The spectrum on the bottom is that of compound **2** and that on the top is the crude material obtained from the aforementioned treatment. (B) Full HPLC traces of compound **2** hydrolysis carried out chemically at pH = 14 and in PBS/mouse plasma as noted above. This is the full trace corresponding to the edited section shown in Figure 2 in the main paper.

Chemistry

General Information. All chemicals were used as received from a commercial source without further purification. All reactions were performed in round-bottom flasks sealed with rubber septa or glass vials sealed with PTFE/silicone septa equipped polypropylene caps. Plastic syringes or glass pipets were used to transfer liquid reagents. Reactions were stirred magnetically using Teflon-coated, magnetic stir bars. Reactions were monitored by UPLC-UV-LRMS. Compounds were synthesized according to the procedure of Gold, 2016.[1] Purification was carried out on an automated flash chromatography/medium-pressure liquid chromatography (MPLC) system using normal-phase silica flash columns (4, 12, 24 or 40g) or reverse-phase C-18 columns (15, 50,150 g). All nuclear magnetic resonance spectra were recorded on a 400 MHz or 600 MHz with a dual carbon/proton cryoprobe instrument. NMR samples were recorded in $CDCl_3$ or DMSO-d_6. Chemical shifts are reported in parts per million (ppm) and are referenced to the center line of the solvent (for $CDCl_3$, δ 7.26 ppm for ^{1}H NMR and 77.0 for ^{13}C NMR; for DMSO-d_6, δ 2.50 ppm for ^{1}H NMR and 39.5 for ^{13}C NMR).Coupling constants are given in hertz (Hz). HRMS data were collected with time-of-flight (TOF) or high-field orbitrap (HF-X) and an electrospray ion source. Purity of all compounds was analyzed using Waters UPLC or Agilent 1200 RRLC under acidic or basic mobile phase. Spectroscopic data for the known compounds prepared according to the methodology described in the paper match with those reported in the literature.

Except where noted, compounds were prepared as reported by Gold et al.[1]

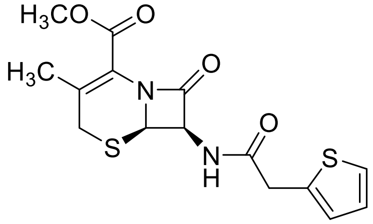

Methyl (6*R*,7*R*)-3-methyl-8-oxo-7-(2-(thiophen-2-yl)acetamido)-5-thia-1-azabicyclo[4.2.0]oct-2-ene-2-carboxylate (3a). IR (film) ν_{max} = 3281, 1765, 1722, 1647 cm^{-1}; ^{1}H NMR (DMSO-d_6, 400 MHz): δ 9.09 (d, J = 8.2 Hz, 1H), 7.36 (dd, J = 5.0, 1.4 Hz, 1H), 6.94 (m, 2H), 5.63 (dd, J = 8.2, 4.6 Hz, 1H), 5.07 (d, J = 4.6 Hz, 1H), 3.75 (m, 5H), 3.50 (AB q, $\Delta\nu_{AB}$ = 74.5 Hz, J_{AB} = 19.2 Hz, 2H), 2.02 (s, 3H); ^{13}C NMR (DMSO-d_6, 101 MHz): δ 169.9, 164.5, 162.5, 136.9, 131.3, 126.6, 126.3, 125.0, 121.5, 59.0, 57.2, 52.1, 35.7, 29.0, 19.4; HRMS (ESI-TOF): calcd for $C_{15}H_{17}N_2O_4S_2$ [M+H]$^+$ 353.0624; found, 353.0639. Purity (HPLC): 99%.

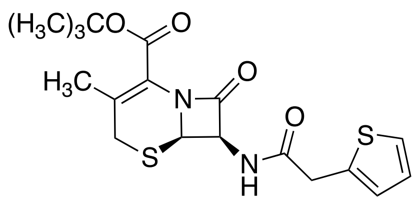

***tert*-butyl (6*R*,7*R*)-3-methyl-8-oxo-7-(2-(thiophen-2-yl)acetamido)-5-thia-1-azabicyclo[4.2.0]oct-2-ene-2-carboxylate (3b).** *tert*-Butyl (6*R*,7*R*)-7-amino-3-methyl-8-oxo-5-thia-1-azabicyclo[4.2.0]oct-2-ene-2-carboxylate[2] was acylated with 2-(thiophen-2-yl)acetyl chloride in 2:1 CH_2Cl_2/aq satd $NaHCO_3$. IR (film) ν_{max} = 3281, 1768, 1714, 1664 cm^{-1}; ^{1}H NMR (DMSO-d_6, 400 MHz): δ 9.08 (d, *J* = 8.3 Hz, 1H), 7.36 (dd,*J* = 5.1, 1.4 Hz, 1H), 6.94 (m, 2H), 5.60 (dd, *J* = 8.3, 4.7 Hz, 1H), 5.05 (d, *J* = 4.7 Hz, 1H), 3.76 (s, 2H), 3.46 (AB q, $\Delta\nu_{AB}$ = 90.2 Hz, J_{AB} = 18.0 Hz, 2H), 1.97 (s, 3H), 1.46 (s, 9H); ^{13}C NMR (DMSO-d_6, 101 MHz): δ 169.9, 164.5, 162.5, 136.9, 131.3, 126.6, 126.3, 125.0, 121.5, 59.0, 57.2, 52.1, 35.7, 29.0, 19.4; HRMS (ESI-TOF): calcd for $C_{18}H_{23}N_2O_4S_2$ $[M+H]^+$ 395.1094; found, 395.1074. Purity (HPLC): 100%.

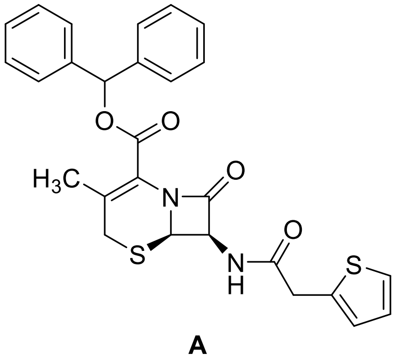

A

and

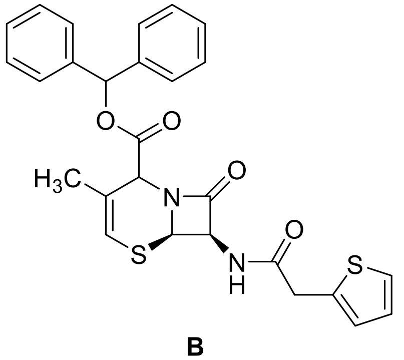

B

Benzhydryl (6*R*,7*R*)-3-methyl-8-oxo-7-(2-(thiophen-2-yl)acetamido)-5-thia-1-azabicyclo[4.2.0]oct-2-ene-2-carboxylate (3c) and isomer. Benzhydryl (6*R*,7*R*)-7-amino-3-methyl-8-oxo-5-thia-1-azabicyclo[4.2.0]oct-2-ene-2-carboxylate[3] was acylated with 2-(thiophen-2-yl)acetyl chloride in CH_2Cl_2 with triethylamine at 0 °C. Mixture of isomers, A:B 1.6:1. IR (film) ν_{max} = 3291, 1771, 1669, 1533 cm^{-1}; ^{1}H NMR ($CDCl_3$, 400 MHz): major isomer δ 7.33 (m, 12H), 7.36 (dd, *J* = 5.1, 1.4 Hz, 1H), 6.98 (m, 2H), 6.90 (s, 1H) 6.45 (d, *J* = 9.1 Hz, 1H), 5.79 (dd, *J* = 9.0, 4.7 Hz, 1H), 4.95 (d, *J* = 4.7 Hz, 1H), 3.85 (s, 2H), 3.28 (AB q, $\Delta\nu_{AB}$ = 99.6 Hz, J_{AB} = 19.6 Hz, 2H), 2.09 (s, 3H), minor isomer (diagnostic peaks only) δ 6.88 (s, 1H) 6.41 (d, *J* = 8.4 Hz, 1H), 5.88 (t, *J* = 1.6 Hz, 1H), 5.63 (dd, *J* = 9.2, 4.1 Hz, 1H), 5.21 (d, *J* = 4.0 Hz, 1H), 4.80 (s, 1H), 3.84 (s, 2H), 1.78 (s, 3H); ^{13}C NMR ($CDCl_3$, 101 MHz): major isomer (diagnostic peaks only) δ 170.0, 164.5, 161.2, 133.6, 79.0, 58.9, 57.2, 37.11, 30.3, 20.2; minor isomer (diagnostic peaks only) δ 169.7, 166.3, 164.7, 78.9, 59.9, 53.2, 52.7, 37.12, 22.1. It was not possible to unambiguously assign aromatic signals to the major or minor isomers. The aromatic portion of the spectrum is depicted below. HRMS (ESI-TOF): calcd for $C_{27}H_{25}N_2O_4S_2$ $[M+H]^+$ 505.1256; found, 505.1233. Purity (HPLC): 98%.

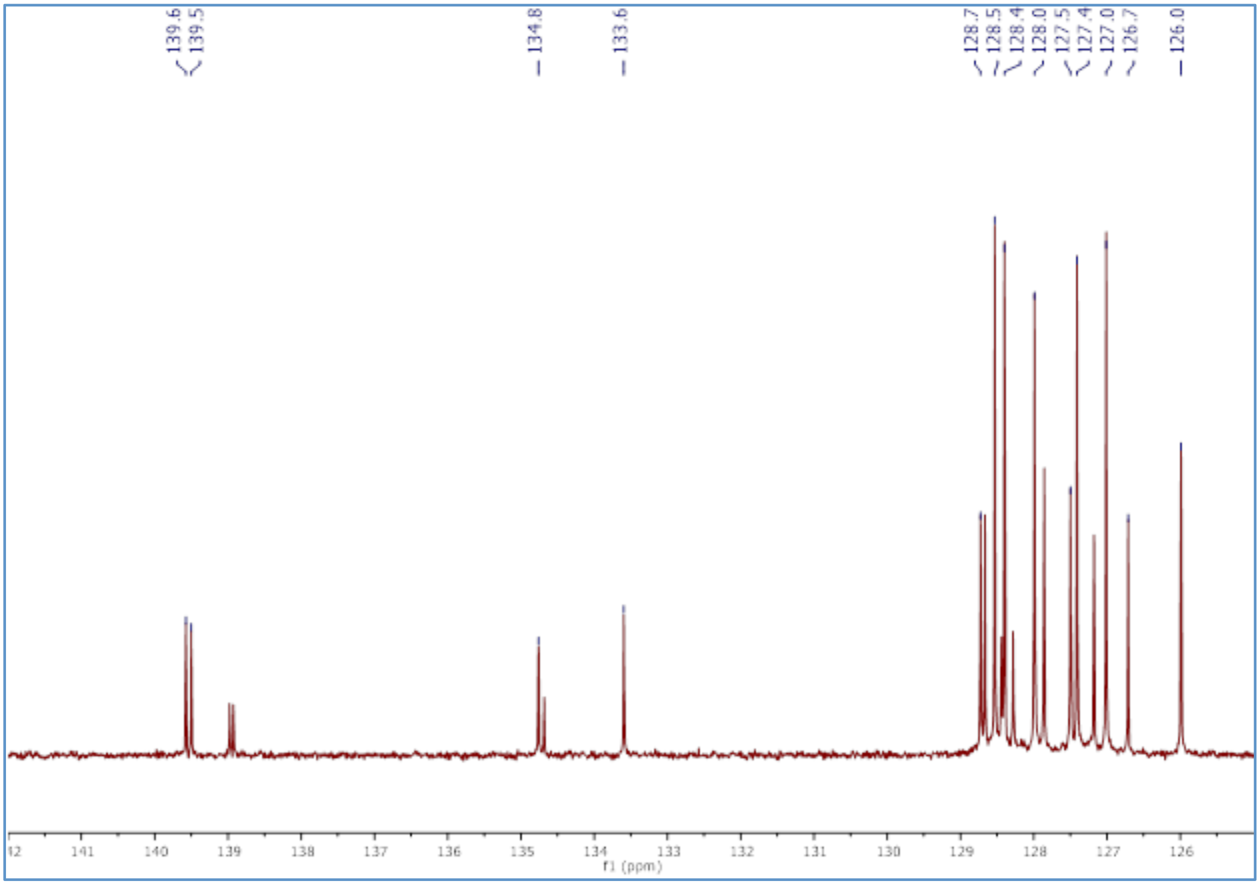

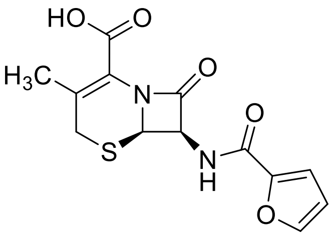

(6*R*,7*R*)-7-(Furan-2-carboxamido)-3-methyl-8-oxo-5-thia-1-azabicyclo[4.2.0]oct-2-ene-2-carboxylic acid (4a). IR (film) ν_{max} = 3297, 1738, 1720, 1682 cm^{-1}; ^1H NMR (DMSO-d_6, 600 MHz): δ 13.16 (bs, 1H), 9.23 (d,*J* = 8.2 Hz, 1H), 7.89 (dd, *J* = 1.8, 0.8 Hz, 1H), 7.36 (dd, *J* = 3.5, 0.8 Hz, 1H), 6.64 (dd, *J* = 3.5, 1.8 Hz, 1H), 5.69 (dd, *J* = 8.1, 4.6 Hz, 1H), 5.10 (d, *J* = 4.6 Hz, 1H), 3.47 (AB q, $\Delta\nu_{AB}$ = 71.8 Hz, J_{AB} = 19.2 Hz, 2H), 2.03 (s, 3H); ^{13}C NMR (DMSO-d_6, 151 MHz): δ 163.6, 158.0, 146.5, 146.0, 130.9, 123.1, 114.9, 111.9, 59.0, 57.5, 29.1, 19.4.; HRMS (ESI-HF-X): calcd for $C_{13}H_{11}N_2O_5S$ [M-H]$^-$ 307.03942; found, 307.03988. Purity (HPLC): 99%.

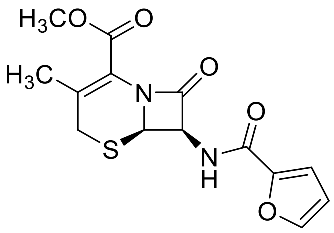

Methyl (6*R*,7*R*)-7-(furan-2-carboxamido)-3-methyl-8-oxo-5-thia-1-azabicyclo[4.2.0]oct-2-ene-2-carboxylate (4b). IR (film) ν_{max} = 3319, 1769, 1720, 1662 cm^{-1}; ^{1}H NMR (DMSO-d_6, 400 MHz): δ 7.44 (dd, J = 1.8, 0.8 Hz, 1H), 7.26 (d, J = 9.0 Hz, 1H), 7.13 (dd, J = 3.5, 0.8 Hz, 1H), 6.46 (dd, J = 3.5, 1.8 Hz, 1H), 5.88 (dd, J = 9.0, 4.7 Hz, 1H), 4.99 (d, J = 4.7 Hz, 1H), 3.78, (s, 3H), 3.34 (AB q, $\Delta\nu_{AB}$ = 93.1 Hz, J_{AB} = 19.2 Hz, 2H), 2.10 (s, 3H); ^{13}C NMR (DMSO-d_6, 101 MHz): δ 164.2, 162.4, 157.9, 146.4, 144.7, 132.2, 122.2, 115.5, 112.1, 58.5, 57.2, 52.2, 30.0, 19.8; HRMS (ESI-HF-X): calcd for $C_{14}H_{14}N_2O_5SNa$ $[M+Na]^+$ 345.05156; found, 345.05077. Purity (HPLC): 99%.

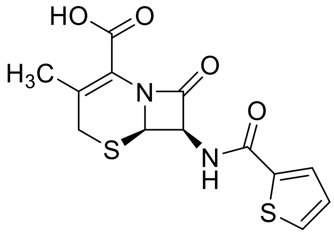

(6*R*,7*R*)-3-Methyl-8-oxo-7-(thiophene-2-carboxamido)-5-thia-1-azabicyclo[4.2.0]oct-2-ene-2-carboxylic acid (5a). IR (film) ν_{max} = 3319, 1739, 1724, 1664 cm^{-1}; ^{1}H NMR (DMSO-d_6, 400 MHz): δ 13.04 (bs, 1H), 9.38 (d, J = 8.0 Hz, 1H), 8.00 (dd, J = 3.8, 1.1 Hz, 1H), 7.83 (dd, J = 5.0, 1.1 Hz, 1H), 7.17 (dd, J = 5.0, 3.7 Hz, 1H), 5.72 (dd, J = 8.0, 4.6 Hz, 1H), 5.11 (d, J = 4.6 Hz, 1H), 3.48 (AB q, $\Delta\nu_{AB}$ = 63.4 Hz, J_{AB} = 18.8 Hz, 2H), 2.03 (s, 3H); ^{13}C NMR (DMSO-d_6, 101 MHz): δ 163.6, 163.5, 161.6, 138.2, 132.0, 130.1, 129.7, 128.1, 123.1, 59.5, 57.5, 29.0, 19.4.; HRMS (ESI-HF-X): calcd for $C_{13}H_{11}N_2O_4S_2$ $[M-H]^-$ 323.01657; found, 323.01725. Purity (HPLC): 96%.

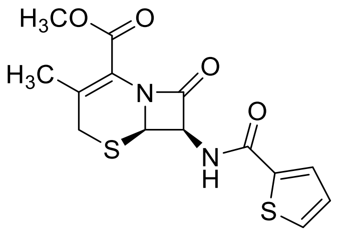

Methyl (6*R*,7*R*)-3-methyl-8-oxo-7-(thiophene-2-carboxamido)-5-thia-1-azabicyclo[4.2.0]oct-2-ene-2-carboxylate (5b). IR (film) ν_{max} = 3332, 1761, 1722, 1634 cm^{-1}; ^{1}H NMR (DMSO-d_6, 600 MHz): δ 9.40 (d, *J* = 8.0 Hz, 1H), 8.00 (dd, *J* = 3.8, 1.2 Hz, 1H), 7.83 (dd, *J* = 5.0, 1.1 Hz, 1H), 7.17 (dd, *J* = 5.0, 3.7 Hz, 1H), 5.76 (dd, *J* = 8.0, 4.6 Hz, 1H), 5.15 (d, *J* = 4.6 Hz, 1H), 3.76, (s, 3H), 3.52 (AB q, $\Delta\nu_{AB}$ = 90.0 Hz, J_{AB} = 18.0 Hz, 2H), 2.03 (s, 3H); ^{13}C NMR (DMSO-d_6, 151 MHz): δ 162.5, 161.5, 138.2, 132.0, 131.5, 129.7, 128.2, 121.8, 59.6, 57.6, 52.1, 29.1, 19.4.; HRMS (ESI-HF-X): calcd for $C_{14}H_{14}N_2O_4S_2Na$ [M+Na]$^+$ 361.02872; found, 361.02793. Purity (HPLC): 98%.

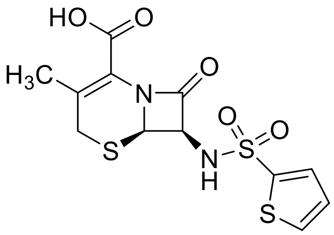

(6*R*,7*R*)-3-Methyl-8-oxo-7-(thiophene-2-sulfonamido)-5-thia-1-azabicyclo[4.2.0]oct-2-ene-2-carboxylic acid (6a). IR (film) ν_{max} = 1760, 1701, 1343 cm^{-1}; ^{1}H NMR (DMSO-d_6, 600 MHz): δ 13.16 (bs, 1H), 9.32 (d, *J* = 9.2 Hz, 1H), 7.95 (dd, *J* = 5.0, 1.4 Hz, 1H), 7.68 (dd, *J* = 3.7, 1.4 Hz, 1H), 7.19 (dd, *J* = 5.0, 3.7 Hz, 1H), 5.32 (dd, *J* = 7.0, 3.6 Hz, 1H), 4.95 (d, *J* = 4.6 Hz, 1H), 3.40 (AB q, $\Delta\nu_{AB}$ = 108.9 Hz, J_{AB} = 18.0 Hz, 2H), 1.99 (s, 3H); ^{13}C NMR (DMSO-d_6, 151 MHz): δ 163.4, 163.2, 141.4, 133.0, 132.0, 130.5, 127.8, 122.6, 61.8, 56.0, 29.0, 19.4; HRMS (ESI-HF-X): calcd for $C_{12}H_{11}N_2O_5S_3$ [M-H]$^-$ 358.98356; found, 358.98483. Purity (HPLC): 95%.

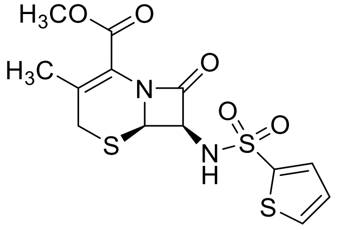

Methyl (6*R*,7*R*)-3-methyl-8-oxo-7-(thiophene-2-sulfonamido)-5-thia-1-azabicyclo[4.2.0]oct-2-ene-2-carboxylate (6b). IR (film) ν_{max} = 3246, 1766, 1719, 1347 cm^{-1}; ^{1}H NMR (CDCl$_3$, 400 MHz): δ 7.68 (dd, *J* = 3.8, 1.4 Hz, 1H), 7.62 (dd, *J* = 5.0, 1.3 Hz, 1H), 7.10 (dd, *J* = 5.0, 3.8 Hz, 1H), 6.05 (d, *J* = 9.7 Hz, 1H), 5.22 (dd, *J* = 9.8, 4.6 Hz, 1H), 4.87 (d, *J* = 4.6 Hz, 1H), 3.78, (s, 3H), 3.30 (AB q, $\Delta\nu_{AB}$ = 88.3 Hz, J_{AB} = 18.4 Hz, 2H), 2.03 (s, 3H); ^{13}C NMR (DMSO-d_6, 101 MHz): δ 163.5, 162.4, 141.4, 133.1, 132.0, 131.9, 127.8, 121.4, 61.9, 57.4, 52.1, 29.1, 19.4.; HRMS (ESI-HF-X): calcd for $C_{13}H_{14}N_2O_5S_3Na$ [M+Na]$^+$ 396.99571; found, 396.99499. Purity (HPLC): 93%.

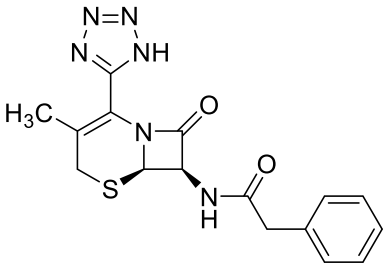

***N*-((6*R*,7*R*)-3-Methyl-8-oxo-2-(1H-tetrazol-5-yl)-5-thia-1-azabicyclo[4.2.0]oct-2-en-7-yl)-2-phenylacetamide (7)**, Compound prepared from (6*R*,7*R*)-7-amino-3-methyl-8-oxo-5-thia-1-azabicyclo[4.2.0]oct-2-ene-2-carboxylic acid (7-ADCA) according to reported procedures.[4,5] IR (film) ν_{max} = 1786, 1645, 1541, 1348 cm^{-1}; ^{1}H NMR (DMSO-d_6, 600 MHz): δ 9.15 (d, 8.30 Hz, 1H), 7.27 (m, 5H), 5.62 (q, *J* = 4.4 Hz, 1H), 5.24 (d, *J* = 4.7 Hz, 1H), 3.68 (d, *J* = 18.0 Hz, 1H), 3.54 (m, 3H), 2.02 (s, 3H); ^{13}C NMR (DMSO-d_6, 151 MHz): δ 171.04, 170.95, 165.0, 135.77, 135.75, 129.0, 128.2, 126.5, 115.3 (vbr), 59.0, 58.9, 57.6, 41.6, 41.5, 28.5,19.3; HRMS (ESI-HF-X): calcd for $C_{16}H_{15}N_6O_2S$ $[M-H]^-$ 355.09827; found, 355.09915. Purity (HPLC): 100%.

References

1. Gold, B.; Smith, R.; Nguyen, Q.; Roberts, J.; Ling, Y.; Lopez Quezada, L.; Somersan, S.; Warrier, T.; Little, D.; Pingle, M.; Zhang, D.; Ballinger, E.; Zimmerman, M.; Dartois, V.; Hanson, P.; Mitscher, L. A.; Porubsky, P.; Rogers, S.; Schoenen, F. J.; Nathan, C.; Aubé, J. *J. Med. Chem.* **2016**, *59*, 6027-6044.
2. Grigan, N.; Musel, D.; Veinberg, G. A.; Lukevics, E. *Synth. Comm*. **1996**, *26*, 1183-1185
3. Micetich, R. G.; Singh, R. *Synthesis* **1985**, 693-695.
4. Barth, W. E. US Patent 4,039,533, February 17, 1976.
5. Nishitani, Y.; Aoki, T.; Yamawaki, K.; Yokoo, K.; Sano, M. US Patent 2014/0088302 A1, April 25, 2012.
